# Integrated use of phosphorus fertilizer and farmyard manure improves wheat productivity by improving soil quality and P availability in calcareous soil under subhumid conditions

**DOI:** 10.3389/fpls.2023.1034421

**Published:** 2023-01-23

**Authors:** Aftab Jamal, Muhammad F. Saeed, Adil Mihoub, Bryan G. Hopkins, Iftikhar Ahmad, Asif Naeem

**Affiliations:** ^1^ Department of Soil and Environmental Sciences, Faculty of Crop Production Sciences, University of Agriculture, Peshawar, Pakistan; ^2^ Department of Environmental Science, COMSATS University, Islamabad, Vehari Campus, Vehari, Pakistan; ^3^ Center for Scientific and Technical Research on Arid Regions, Biophysical Environment Station, Touggourt, Algeria; ^4^ Department of Plant and Wildlife Sciences, Brigham Young University, Provo, Utah, United States; ^5^ Soil and Environmental Sciences Division, Nuclear Institute for Agriculture and Biology, Faisalabad, Pakistan

**Keywords:** manure, phosphorus, wheat, calcareous, phosphorus absorption efficiency, phosphorus balance, phosphorus uptake, phosphorus use efficiency

## Abstract

**Introduction:**

Low soil fertility and high fertilizer costs are constraints to wheat production, which may be resolved with integrating fertilizer phosphorus (P) and farm-yard manure (FYM). Study objectives were to evaluate P source impacts on soil, P efficiency, and wheat growth in a calcareous soil.

**Methods:**

Treatments included P fertilizer (0, 17, 26, or 39 kg P ha-1) and/or FYM (0 or 10 T ha-1) in a: 1) incubation experiment and 2) wheat (Triticum aestivum spp.) field experiment.

**Results and Discussion:**

Soil organic matter increased (30-72%) linearly for both fertilizer and FYM, whereas pH decreased (0.1-0.3 units) with fertilizer only. Addition of fertilizer and FYM increased plant available P (AB-DTPA extractable soil P) an average of 0.5 mg P kg-1 soil week-1 with incubation. The initial increase was 1-9 mg P kg-1, with further increase after 84 d of ~3-17 mg P kg-1. There was also a significant increase of available P in the soil supporting plants in the field study, although the magnitude of the increase was only 2 mg kg-1 at most for the highest fertilizer rate + FYM. Grain (66 to 119%) and straw (25-65%) yield increased significantly, peaking at 26 kg P ha-1 + FYM. The P Absorption Efficiency (PAE), P Balance (PB), and P Uptake (PU) increased linearly with P rate, with the highest levels at the highest P rate. The P Use Efficiency (PUE) was highest at the lowest rates of P, with general decreases with increasing P, although not consistently. Principal component analysis revealed that 94.34 % of the total variance was accounted for with PC1 (84.04 %) and PC2 (10.33 %), with grain straw yield significantly correlated to SOM, PU, and PAE. Regression analysis showed highly significant correlation of PB with P-input (R2= 0.99), plant available P (R2= 0.85), and PU (R2= 0.80). The combination of FYM at the rate of 10 T ha-1 and fertilizer P at 26 kg P ha-1 was found as the optimum dose that significantly increased yield. It is concluded that FYM concoction with fertilizer-P not only improved SOM and residual soil P, but also enhanced wheat yields with reasonable P efficiency.

## 1 Introduction

Wheat (*Triticum aestivum* L.) is the world’s leading agronomic crop in production value and acreage (Hopkins and Hansen, 2019). Wheat is Pakistan’s most important cereal crop, accounting for 8.7% of agricultural value addition and 1.7% of the gross domestic product (GDP) (Khan et al., 2022).

Although Pakistan’s soil and climate conditions are favorable and high-yielding cultivars are available, wheat grain production is reduced due to the calcareous nature of most Pakistani soils and poor nutrient management, particularly that of phosphorus (P) (Ul-Allah et al., 2018). Low soil fertility due to continuous cropping, with little or no external inputs and crop residue removal, are other causes of low production (NFDC, 2001).

Plants require P as an essential macronutrient to complete their life cycles (Marschner, 2012; Hopkins, 2015; Ding et al., 2020; Hopkins, 2020). In calcareous soils, a majority of applied P fertilizer is adsorbed on the calcite surface and becomes temporarily unavailable to plants, which can cause a yield reduction (Hopkins, 2015; Saeed et al., 2021). Plant-available P in soil is affected by soil chemistry properties, especially pH and limestone content (Lindsay, 2001; Fixen and Bruulsema, 2014). Calcareous soils are commonly deficient in plant-available P due to poor solubility as a result of fixation and sorption (Lindsay, 2001; Manzoor, 2013; Hopkins et al., 2014; Mihoub and Boukhalfa-Deraoui, 2014; Deraoui et al., 2015; Hopkins, 2020). The resulting effect of low P solubility is relatively poor fertilizer P efficiency (Jamal et al., 2018; Mihoub et al., 2019). Thus, a great majority of calcareous soils need relatively high amounts of extraneous supplementation of P for sustained crop yield (Fixen and Bruulsema, 2014; Hopkins et al., 2014; Hopkins and Hansen, 2019; Pradhan et al., 2021). Therefore, P dynamics in these soils must be known to evaluate their availability to plants (Manzoor, 2013).

Furthermore, the present price boost in P fertilizers is reflected in its decreased application to crops by resource-poor farmers, potentially resulting in reduced crop production (Aboukila et al., 2018). In some regions, including Pakistan, it is difficult to convince the farming community to apply full-recommended fertilizer doses for wheat, but it seems possible to improve fertilizer P use efficiency (PUE) in calcareous soils by selecting efficient P sources and adopting appropriate time and methods of application. To address the aforementioned issues, cost-effective, environmentally friendly, and more productive farming technologies must be developed (Hopkins, 2015; Mihoub et al., 2019; Hopkins, 2020).

Yields have steadily increased since the onset of the Green Revolution, and as a result, there is an increasing need to efficiently supply P to plants while minimizing negative impacts on the environment (Hopkins and Hansen, 2019; Khan et al., 2022). Many studies have shown that organic manures may partially or entirely substitute chemical fertilizers, reducing dependency on limited rock phosphate reserves (Khan et al., 2022; Mihoub et al., 2022). Manure (commonly referred to as “farmyard manure” (FYM) in some regions, including Pakistan) is less concentrated, and P is bound to various molecules that must be decomposed to convert it to inorganic phosphates that plants can take up (Hopkins, 2015; Mihoub et al., 2022). However, an advantage of the FYM is that it also contains all other essential plant nutrients, which can reduce the possibility of P-induced deficiencies of other nutrients (Barben et al., 2011). It is also considered a slow-release source of P (Hopkins, 2015). The FYM also contains a wide variety of other molecules, including organic acids, that can be beneficial as they improve P movement through soil and bioavailability (Hill et al., 2015a; Hill et al., 2015b; Hopkins, 2015; Summerhays et al., 2015). The addition of soil organic matter (SOM) may improve soil chemical, physical, and biological properties, which can then positively impact nutrient cycling and provide an enhanced environment for vegetation growth (Wu et al., 2013).

As previously mentioned, animal manures are considered a valuable nutrient source when applied to the soil with proper management (Pradhan et al., 2021), although it has also been reported that the sole application of organic amendments could hamper nutrient availability due to fixation (Hazra et al., 2018). Its decomposition rate is relatively faster than many other organic nutrient sources (Rehim et al., 2020), but this decomposition can lead to increases in SOM. This process can play a prominent role in improving soil structure, which in turn provides favorable environments for root development (Zhang et al., 2014) and improves soil water-holding capacity (Wu et al., 2013). It has been reported as a valuable fertilizer for wheat (*Triticum aestivum* L.) production by increasing SOM content (Rehim et al., 2020).

In this regard, the integration of traditional chemical P fertilizers and organic amendments, such as FYM, could be a possible option for improving the efficiency of fertilizer P use in highly calcareous P-sorbing soils (Hopkins, 2015). Although there are many studies examining chemical fertilizer and manure P in wheat, there needs to be an evaluation of the changes in P transformations and grain yield of bread wheat induced by the combined application of FYM and chemical P fertilizer. Moreover, it needs to be clarified how P fertilizer in combination with FYM may affect nutrient balance and the need for, or not, chemical fertilizers in calcareous soils. This point is considered one of the most important recent trends in studies related to soil quality and soil-plant relations regarding P under these conditions.

Therefore, studies were conducted to determine whether the combination of P fertilizer with FYM in calcareous soils under subhumid climatic conditions found in Pakistan can: (1) improve soil properties and P availability; (2) improve phosphorus absorption efficiency (PAE), phosphorus balance (PB), phosphorus uptake (PU), and PUE in wheat; and (3) increase wheat productivity. For this study, it was hypothesized that the application of inorganic P in combination with FYM improves wheat grain yield by improving soil properties, PAE, PB, PU, and PUE in calcareous soils.

## 2 Materials and methods

### 2.1 Experimental site and soil characteristics

The experiment was carried out at a greenhouse and a field location immediately next to each other located at the Institute of Biotechnology and Genetic Engineering at the University of Agriculture, Peshawar, Pakistan (34° 01′, 14.2″ N and 71° 28′, 52.6″ E). The preceding crop in the location was maize (*Zea mays* L.). This region lies 340 m above sea level and is classified as a warm-temperate zone. The average annual temperature is ~22°C, with the highest average in June at ~33°C and the lowest in January at ~10°C. The average annual precipitation is 640 mm, with the least amount of rainfall occurring in November.

A composite soil sample was collected at 0–20 cm depth before the experiment with the following results: pH = 8.4 (Richards, 1954), salinity as electrical conductivity (EC) = 0.25 dS m^−1^ (Richards, 1954), SOM = 5.9 g kg^−1^ (Nelson and Sommers, 1982), total nitrogen (N) = 5 mg kg^−1^, and plant-available P and potassium (K) = 4.7 and 130 mg kg^−1^, respectively, as determined through extraction with ammonium bicarbonate-diethylenetriamine pentaacetate (AB-DTPA) (Soltanpour, 1985).

### 2.2 Experimental design and measurements

#### 2.2.1 Treatments: Laboratory incubation and field experiment

Four chemical P fertilizer rates were applied without or with 10 T FYM ha^−1^ in both studies (**Table 1**). The chemical fertilizer was single superphosphate (SSP; 8% P). The FYM was derived from well-decomposed cattle (*Bos taurus*) excreta (dung and urine) mixed with some crop residues, such as rice and cotton straw. The physiochemical properties and nutrient constituents of FYM were as follows: brown to black color, pH = 8.0, EC = 2.1 dS m^−1^, total N = 13.6 g kg^−1^, total P = 1.5 g kg^−1^, and total K = 8.4 g kg^−1^.

**Table 1:** Phosphorus (P) treatment rates (kg ha^−1^) applied as single superphosphate (SSP) and/or farmyard manure (FYM) for a laboratory and a field experiment.

Treatment	Chemical fertilizer (F)		Manure fertilizer (M)		Total P applied
ID	P (kg ha^−1^)	SSP (kg ha^−1^)	P (kg ha^−1^)	FYM (T ha^−1^)	P (kg ha^−1^)
F_0_M_0_	0	0	0	0	0
F_0_M_15_	0	0	15	10	15
F_17_M_0_	17	220	0	0	17
F_17_M_15_	17	220	15	10	32
F_26_M_0_	26	330	0	0	26
F_26_M_15_	26	330	15	10	41
F_39_M_0_	39	500	0	0	39
F_39_M_15_	39	500	15	10	54

The ID is derived from the rates of SSP fertilizer (F) at 0, 17, 26, or 39 kg P ha^−1^ and manure (M) at 0 or 10 T FYM ha^−1^ with 0 or 15 kg P ha^−1^, respectively (FYM = 0.15% P).

#### 2.2.2 Soil P release dynamics and mineralization potential: Laboratory incubation

A laboratory experiment was carried out by applying fertilizer and/or FYM (**Table 1**) to 1 kg of soil in plastic pots with no drainage holes. Before filling the soil in pots, the soil and the fertilizer and/or FYM were thoroughly mixed. Each treatment had three replicates, and the pots were arranged in a completely randomized design (CRD) and incubated under laboratory conditions of a 16-h light and 8-h dark cycle at 25°C ± 1°C and 55%–65% relative humidity for 84 days. Distilled water (DW) was added as needed to maintain adequate soil moisture at near-field capacity.

Triplicate soil samples from each treatment were spectrophotometrically analyzed for plant-available P after 0, 7, 14, 28, 56, and 84 days of incubation. The change in P content (weekly turnover) was calculated by subtracting the initial plant-available P at 0 days from the final P at 84 days and dividing by the total number of weeks (12). Mineralization potential (kg ha^−1^ week^−1^) was calculated by multiplying weekly turnover in milligrams of P per kilogram of soil per week (the assumption is that the weight of 1-ha soil is approximately 2 × 10^6^ kg; Sarir et al., 2006).

#### 2.2.3 P efficiency and yield: Field experiment

A field study was conducted by applying the same fertilizer/FYM treatments as described in experiment 1 (**Table 1**). The experimental field was plowed to a 30-cm depth using a rotavator, and then the SSP and/or FYM were applied to the soil in respective plots, each of 10 m^2^, before crop sowing with tillage. The experimental treatments were arranged in a randomized complete block design (RCBD) with three replicates. The seeds of the wheat variety ATTA-HABIB-2010 were surface disinfected with 1% sodium hypochlorite (NaOCl) to minimize microorganism growth on the seed and then rinsed two to three times with DW. The disinfected seeds were then sown at a seeding rate of 120 kg ha^−1^ with a 25-cm row spacing.

The plots were furrow-irrigated. Fertilizer K was applied as potassium sulfate (K_2_SO_4_) to all plots prior to the first irrigation at 22 kg K ha^−1^. At the first and second irrigations, fertilizer N was split and applied in two equal doses as urea [CO(NH_2_)_2_] to all plots at 110 kg N ha^−1^. The N and K were not balanced, with plots receiving FYM having an additional 136 and 84 kg ha^−1^ of N and K, respectively. However, there was no evidence of deficiency or excess of either of these or other nutrients, and, therefore, it is assumed that these nutrients had no effect on the treatments.

The crop was generally raised using the best agronomic and cultural management practices. The crop was largely free of insect and disease damage. No pesticides were applied. Weeds were pulled by hand as and when required. The wheat was hand-harvested using a sickle.

At maturity, whole plant samples were randomly collected from each plot to determine P concentration and accumulation by wheat plants (Jackson, 1973). The total dry matter yield of each plot was recorded; wheat grains were separated using a micro-plot thresher (Kissan wheat thresher, Gojra, Pakistan), and grain yield was recorded. Postharvest soil samples were collected from each plot and the soil properties measured prior to planting were determined again.

To assess changes in the efficiency of applied P fertilizer in the presence of FYM, P uptake (PU; kg ha^−1^) was calculated by multiplying the nutrient concentration values with the total dry matter yield (Eq. 1).


(1) PU=P content in plant tissue ×total plant dry matter

In addition, the percent P uptake efficiency (PUE) was calculated by subtracting the PU in unfertilized soil from fertilized soil and dividing by the P applied (Eq. 2) (Syers et al., 2008).


(2) $$\text{PUE}=\frac{\text{PU in fertilized soil}-\text{PU in unfertilized soil}}{\text{ P applied to the soil}}$$

The PAE (mg mg^−1^) was calculated by dividing PU by available soil P (Eq. 3) (adapted from Castillo et al., 2013):


(3) $$\text{PAE}=\frac{\text{PU}}{\text{Available P }}$$

Finally, the PB (kg ha^−1^) was calculated by subtracting the P output in the harvested wheat crop (measured as PU) from the total P input (P in fertilizer + P in FYM) (Eq. 4) (Sun et al., 2018).


(4) PB = P input − PU

### 2.3 Statistical analysis

The experimental data were statistically evaluated using the statistical software MSTATC 8.1. A two-way ANOVA (in randomized blocks) was used to analyze data, considering the application rates of mineral fertilizer (SSP) and organic amendment (FYM) as the two factors. The significant differences between treatments were compared by critical difference at a 5% level of probability using the *F*-test. A principal component analysis (PCA) was done to classify the treatments according to the measured parameters and to identify the parameters that determine yield increases and P efficiency. A cluster analysis (CA) was performed using Ward’s method to determine the most important traits related to grain yield. A simple linear regression was performed to show the relationship of PB with PU, plant-available P (AB-DTPA), and P input (Excel 2007 software). The PCA and CA analyses were performed using the XLSTAT statistical package software (ver. 2022.1.1.1251, Excel Add-ins).

## 3 Results

### 3.1 Weekly turnover and mineralization potential of P: Laboratory incubation

There were several statistical differences in the laboratory incubation trial (**Supplementary Table S1**). In the control treatment (F_0_M_0_), the plant-available P had only minor fluctuations over time (**Table 2**). Initially (day 0 of incubation), there were significant increases (~1–9 mg P kg^−1^) in plant-available P that were mostly proportional to the applied P rate. By the end of the study (84 days), the differences had increased (~3–17 mg P kg^−1^) as the fertilizer and FYM P moved towards equilibrium with the soil.

**Table 2:** Laboratory incubation: effect of fertilizer phosphorus (P) and farmyard manure (FYM) applications on soil AB-DTPA extractable P transformation during 12 weeks of incubation.

Trts	Incubation period (day)						Mean	Net increase	
	0	7	14	28	56	84			
	Plant-available P (AB-DTPA extractable; mg P kg^−1^ soil)								
F_0_M_0_	6.13 ± 0.15 g	6.54 ± 0.55 e	6.38 ± 0.91 e	5.95 ± 0.19 d	6.48 ± 0.57 g	6.56 ± 0.31 g	6.34 ± 0.24 h	0.43d
F_0_M_15_	6.86 ± 0.14 f	7.73 ± 0.05 d	8.17 ± 0.04 d	8.84 ± 0.13 c	8.98 ± 0.22 f	9.14 ± 0.17 f	8.28 ± 0.88 g	2.28c
F_17_M_0_	9.30 ± 0.68 e	8.90 ± 0.27 c	7.55 ± 0.47 d	9.85 ± 0.24 c	10.70 ± 0.52 e	11.52 ± 0.29 e	9.63 ± 1.39 f	2.22c
F_17_M_15_	10.99 ± 0.22 d	9.25 ± 0.32 c	10.44 ± 0.86 c	13.40 ± 0.47 b	14.18 ± 0.19 d	14.48 ± 0.15 d	12.1 ± 2.18 e	3.49bc
F_26_M_0_	12.10 ± 0.10 c	11.02 ± 0.18 b	9.85 ± 0.13 c	13.17 ± 0.26 b	15.31 ± 0.26 c	16.23 ± 0.53 c	12.94 ± 2.46 d	4.13b
F_26_M_15_	13.94 ± 1.0 ab	12.44 ± 10.08 a	13.60 ± 0.39 a	15.89 ± 1.79 a	17.87 ± 0.98 b	18.43 ± 0.61 b	15.36 ± 2.43 b	4.49b
F_39_M_0_	15.21 ± 0.07 a	13.11 ± 0.01 a	12.10 ± 0.10 b	16.25 ± 0.04 a	20.22 ± 0.02 a	23.40 ± 0.01 a	16.71 ± 4.33 a	8.19a
F_39_M_15_	13.27 b ± 0.1 b	11.22 ± 0.01 b	12.38 ± 0.01 b	15.44 ± 0.01 a	18.44 ± 0.01 b	19.12 ± 0.01 b	14.97 ± 3.26 c	5.85b

Means within each column with different letters per column are significantly different at *p*< 0.05. In the treatment (Trts) column, the letters represent fertilizer (F) and FYM (M), while the numbers represent the rate of P in kilograms per hectare. Data are shown as means ± SD (*n* = 3).

**Figure 1:**
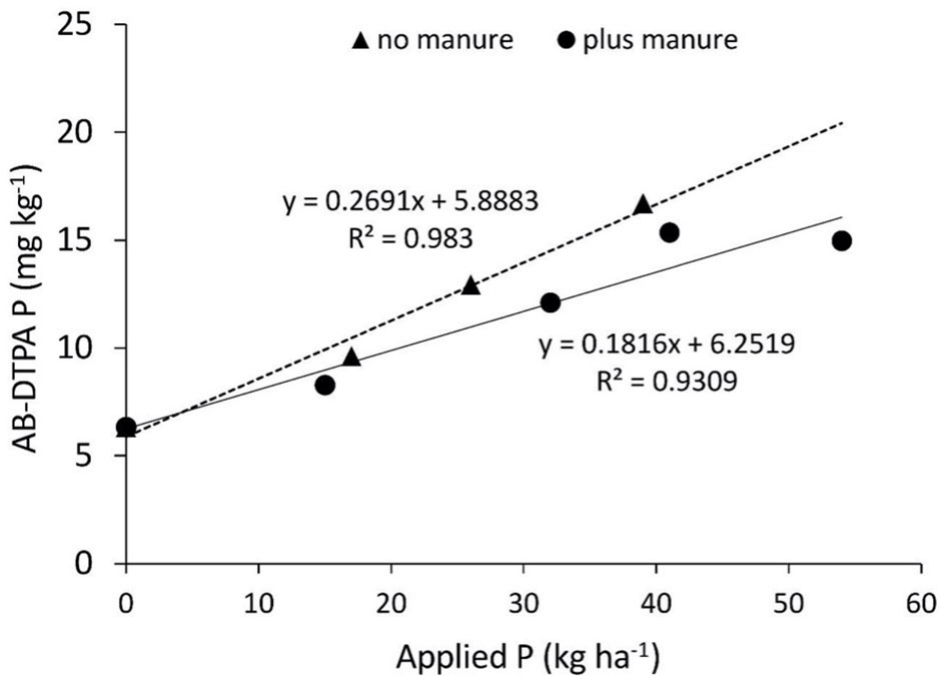
Correlations between plant-available P (extracted with ammonium bicarbonate-diethylenetriamine pentaacetate (AB-DTPA)) and applied P for soil incubated under laboratory conditions. The applied P was chemical fertilizer applied as a single superphosphate (SSP) and/or manure (farmyard manure (FYM)).

The rate of this increase was gradual over the incubation time when the soil was treated with only FYM at 10 T ha^−1^ (F_0_M_15_). The remaining fertilized treatments, regardless of whether they were applied with FYM or not, tended to have a slight decrease in available P during the initial phase of incubation and thereafter an increase. The correlations between available and applied P was very high, especially when no FYM was applied (**Figure 1**).

The application of fertilizer P alone, as well as in conjunction with FYM, significantly affected the weekly turnover and mineralization potential of P in these calcareous soils (**Supplementary Table S2**; **Table 3**). The average change of plant-available P (weekly turnover) with applied P was 0.49 mg P kg^−1^ soil, which was significantly greater than the control. The increase in weekly turnover P against 17 to 26 kg P ha^−1^ was 56% and 19%, respectively. A decline in weekly turnover P was recorded when the soil was treated with the highest fertilizer P at 39 kg P ha^−1^ in conjunction with FYM. The application of FYM significantly increased mineralization potential at 0, 17, 39, and 26 kg P ha^−1^ (**Supplementary Table S3**). A maximum mineralization potential of 32.8 kg P ha^−1^ was recorded in the treatment (F_39_M_0_) followed by treatments (F_39_M_15_) and (F_26_M_15_) with values of 22.5 and 19.5 kg P ha^−1^, respectively.

**Table 3:** Laboratory incubation: effect of fertilizer phosphorus (P) and farmyard manure (FYM) application on weekly turnover P and mineralization potential.

Trts	Weekly P turnover (mg P kg^−1^ soil week^−1^)	Mineralization potential (kg P ha^−1^ season^−1^)
F_0_M_0_	0.03 ± 0.01 f	1.65 ± 0.70 f
F_0_M_15_	0.19 ± 0.01 e	9.12 ± 0.52 e
F_17_M_0_	0.18 ± 0.07 e	8.85 ± 3.59 e
F_17_M_15_	0.29 ± 0.09 d	13.73 ± 0.38 e
F_26_M_0_	0.34 ± 0.03 d	16.49 ± 1.79 d
F_26_M_15_	0.40 ± 0.03 c	19.50 ± 1.82 c
F_39_M_0_	0.68 ± 0.06 a	32.78 ± 0.266 a
F_39_M_15_	0.46 ± 0.03 b	22.49 ± 1.57 b

Means with different letters in the columns are significantly different at *p*< 0.05. The treatments (Trts) with F0, F17, F26, and F39 are 0, 17, 26, or 39 kg ha^−1^ of fertilizer P application, respectively, and with M0 or M15 are 0 and 10 t ha^−1^ of farmyard manure (FYM), respectively (10 T FYM ha^−1^ possesses 0.15% P or 15 kg ha^−1^). Data are shown as means ± SD (*n* = 3).

### 3.2 Straw and grain yields: Field experiment

**Figure 2:**
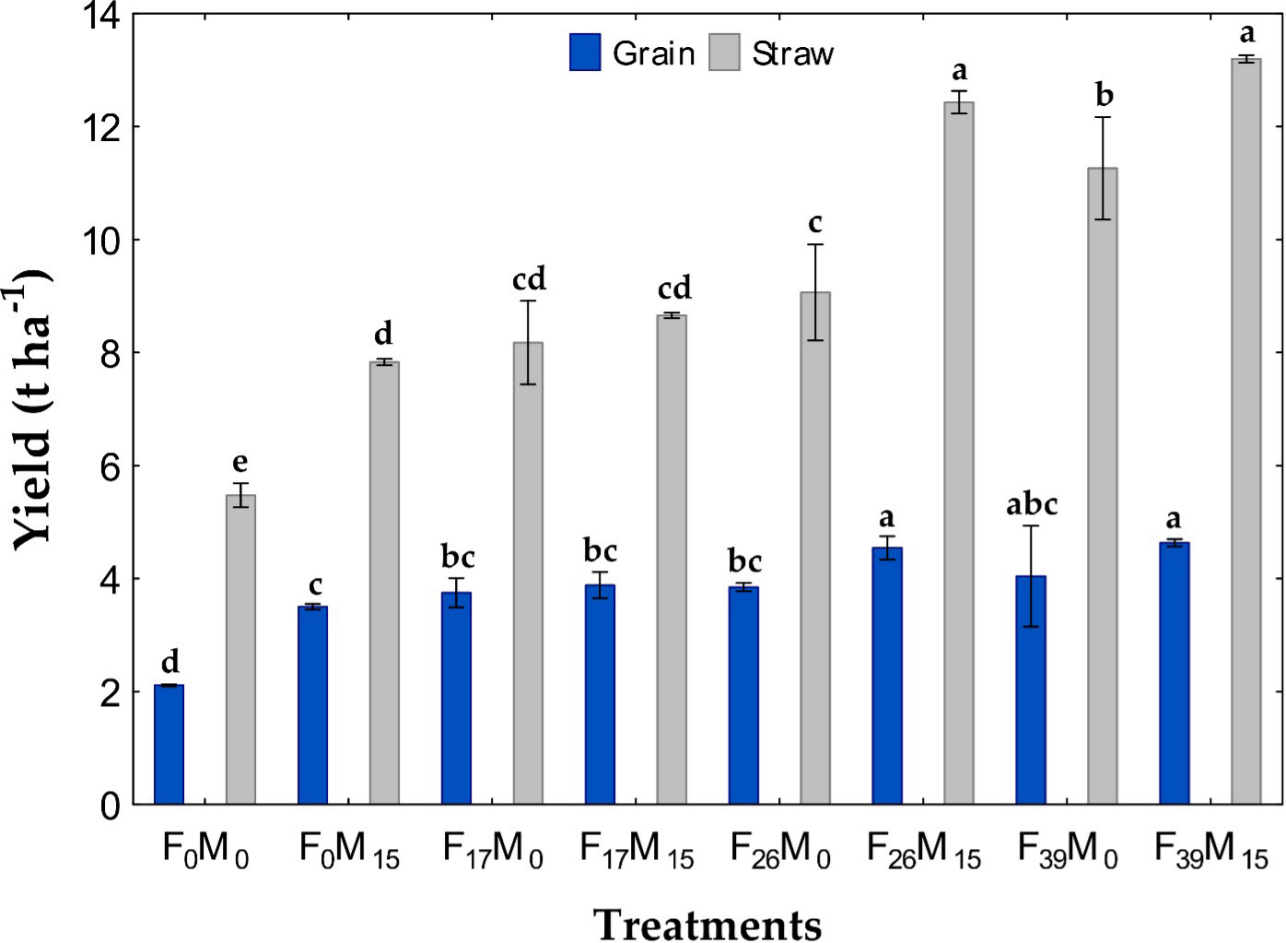
Wheat grain and straw yield as affected by P application from fertilizer **(F)** and/or farmyard manure (FYM). Fertilizer was applied at 0, 17, 26, or 39 kg P ha^−1^, and FYM was applied at 0 or 10 T ha^−1^ with 0 or 15 kg P ha^−1^, respectively (FYM = 0.15% P). Treatment means with different letters are significantly different at *p<* 0.05. Data are shown as means ± SD (*n* = 3).

There were significant differences in grain and straw yields (**Table 4**) for both P sources, as well as their interaction (fertilizer, FYM, and fertilizer × FYM). There were increases in grain and straw yields over the control for all fertilized treatments (**Figure 2**).

**Table 4:** Field study: Statistics (F-Values with P-value significance indicated) for soil properties, yields, and P efficiency.

SOV	DF	SOM	pH	GY	SY	Soil P	PU	PUE	PAE	PB
Fertilizer (F)	3	4.4^**^	5.4^**^	23.5^**^	133.7^**^	24^**^	348.7^**^	13.7^**^	38.8^**^	4,408.2^**^
Manure (M)	1	5.1^**^	3.5^ns^	24.3^**^	90.8^**^	6.6^*^	292.3^**^	60.7^**^	59^**^	3,485.5^**^
F × M	3	0.3^ns^	0.8^ns^	3.3^*^	7.9^**^	0.9^ns^	45^**^	171.7^**^	17.2^**^	45^**^

SOV, source of variation; DF, degrees of freedom; SOM, soil organic matter; GY, grain yield; SY, straw yield; soil P, plant-available P as measured by AB-DTPA extractable soil P; PU, phosphorus uptake; PUE, P use efficiency; PAE, P acquisition efficiency; PB, P balance. ^*^ *p* < 0.05; ^**^ *p* < 0.01; *ns*, not significant.

A significant increase in grain yield was observed with the addition of fertilizer P alone and in combination with FYM, as compared with the control treatment (**Figure 2**). Maximum grain yield was produced with the highest rate of fertilizer (39 kg P ha^−1^), whether FYM was added or not. However, FYM allowed a statistically equivalent yield where it peaked at the second-highest rate of fertilizer (26 kg P ha^−1^) when FYM was also included, which was higher than the yield at this same rate of fertilizer without FYM and all other lower fertilizer rates. Applications of 26 and 39 kg P ha^−1^, reinforced with 10 T ha^−1^ FYM, increased grain yield by 33% and 28%, respectively, as compared with the same amount of fertilizer P applied alone.

Similarly, the maximum straw yield was achieved with the second-highest fertilizer P rate (26 kg P ha^−1^) with FYM (**Figure 2**). However, this was again statistically similar to that of the highest fertilizer rate (39 kg P ha^−1^).

### 3.3 P efficiency: Field experiment

**Figure 3:**
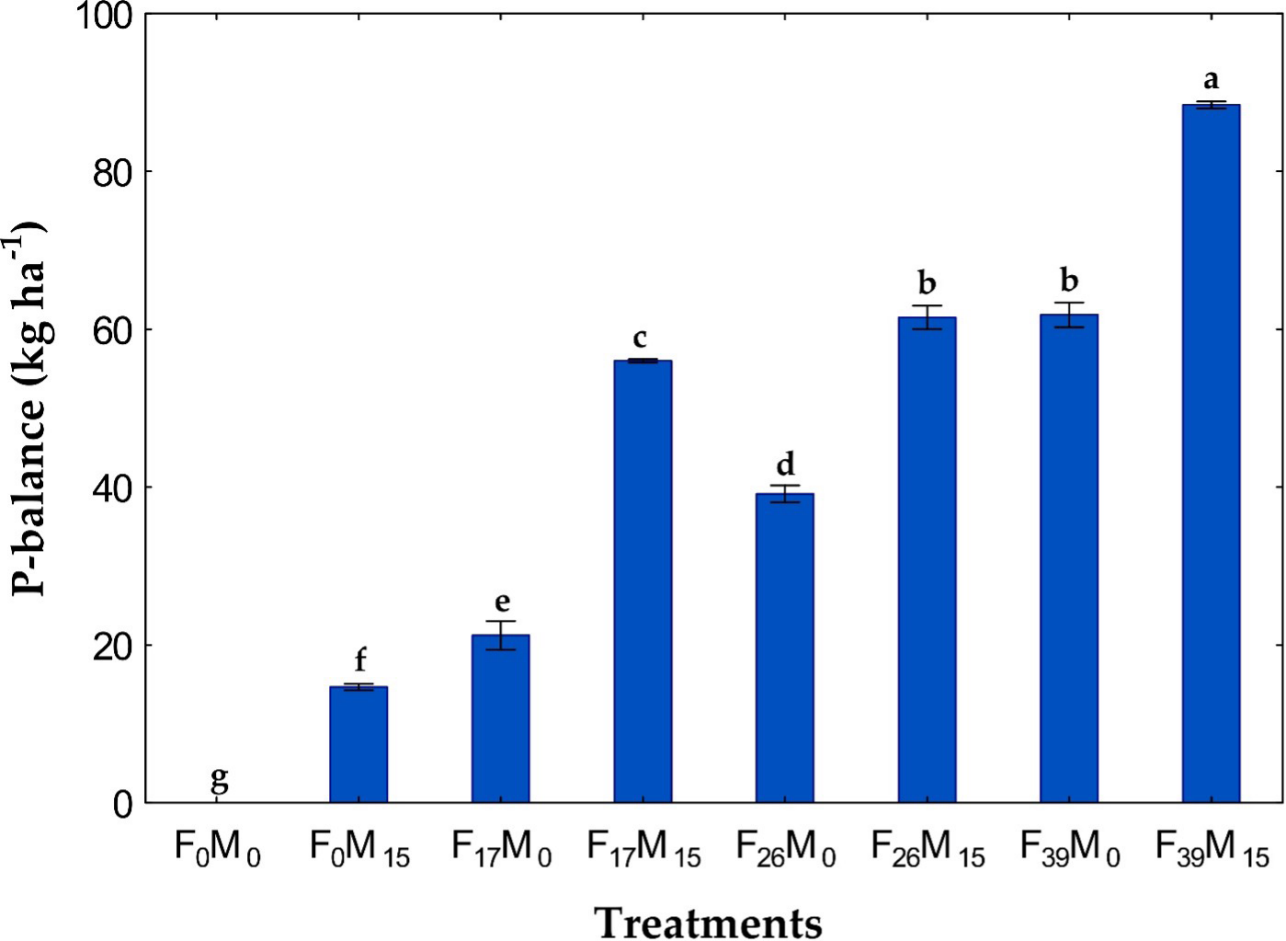
P balance (PB) is affected by P application from fertilizer **(F)** and/or farmyard manure (FYM). Fertilizer was applied at 0, 17, 26, or 39 kg P ha^−1^, and FYM was applied at 0 or 10 T ha^−1^ with 0 or 15 kg P ha^−1^, respectively (FYM = 0.15% P). Treatment means with different letters are significantly different at *p<* 0.05. Data are shown as means ± SD (*n* = 3).

There were significant differences in PU, PB, PUE, and PAE (**Table 4**) for both P sources, as well as their interaction (fertilizer, FYM, and fertilizer × FYM). There were increases in PU, PB, and PAE over the control for all fertilized treatments (**Table 5**; **Figure 3**). As is typical, the PUE was highest at the lowest rates of P (**Table 5**).

**Table 5:** Field study: effect of fertilizer phosphorus (P) and farmyard manure (FYM) application on postharvest plant-available P (AB-DTPA extractable P), P uptake (PU), P use efficiency (PUE), and P acquisition efficiency (PAE).

Trts	AB-DTPA P (mg P kg^−1^ soil)	PU (kg P ha^−1^)	PUE (%)	PAE (mg mg^−1^ soil P)
F_0_M_0_	4.27 ± 0.04 d	7.00 ± 0.2 f	–	1.66 ± 0.04 e
F_0_M_15_	4.48 ± 0.04 cd	19.30 ± 0.3 de	35.6 ± 1.0 a	4.31± 0.08 cd
F_17_M_0_	4.67 ± 0.02 cd	18.70 ± 1.4 de	28.9 ± 3.7 b	4.02 ± 0.29 cd
F_17_M_15_	5.06 ± 0.71 bc	18.00 ± 0.1 e	14.6 ± 0.2 d	3.63 ± 0.52 d
F_26_M_0_	5.29 ± 0.12 b	20.80 ± 0.8 d	22.7 ± 1.4 c	3.94 ± 0.07 cd
F_26_M_15_	6.19 ± 0.004 a	32.40 ± 1.2 b	27.0 ± 1.2 b	5.24 ± 0.19 ab
F_39_M_0_	6.11 ± 0.18 a	28.10 ± 1.2 c	23.2 ± 1.4 c	4.61 ± 0.20 bc
F_39_M_15_	6.35 ± 0.61 a	35.50 ± 0.3 a	22.8 ± 0.2 c	5.65 ± 0.51 a

Means within each column with different letters per column are significantly different at *p*< 0.05. In the treatment (Trts) column, the letters represent fertilizer (F) and FYM (M), while the numbers represent the rate of P in kilograms per hectare. Data are shown as means ± SD (n = 3).

Not surprisingly, the PB, PU, and PAE increased somewhat linearly with the P rate and were significantly highest at the highest P rate (F_39_M_15_) (**Table 5**; **Figure 3**). When comparing with and without FYM, these parameters were higher with FYM at every fertilizer rate except PU and PAE at 17 kg P ha^−1^. It was observed that in the control treatment (F_0_M_0_), the PU exceeded the P additions, leading to a slightly negative PB, whereas for all the other treatments, the PB was positive (**Figure 3**). This indicates residual P in the soil, which may benefit subsequent crops and soil P fertility. The PU followed similar trends as straw yield.

As expected, the maximum PUE was observed with the lowest P fertilizer level with FYM only (F_0_M_15_) as compared to the untreated control and was less for the low fertilizer rate (F_17_M_0_) (**Table 5**). The PUE decreased as fertilizer increased from 17 to 26 kg P ha^−1^ (F_17_M_0_ and F_26_M_0_, respectively). Surprisingly, the PUE did not decrease further at the highest P rate (F_39_M_0_). When FYM was added, the PUE decreased at the low fertilizer rate (F_17_M_0_ vs. F_17_M_15_), but curiously increased at the next highest rate (F_26_M_0_ vs. F_26_M_15_), and there was no difference at the highest rate (F_39_M_0_ vs. F_39_M_15_). Unexpectedly, the lowest PUE was at the lowest fertilizer rate with FYM (F_17_M_15_).

### 3.4 Postharvest soil properties: Field experiment

**Figure 4:**
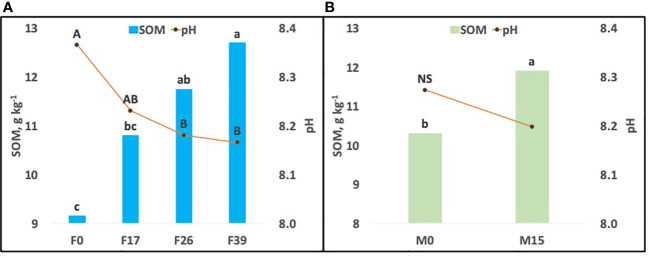
Postharvest soil pH and soil organic matter (SOM) as affected by P application from fertilizer **(F)** and/or farmyard manure (FYM). As the F × FYM interaction was not significant, the fertilizer graph **(A)** was averaged across FYM rates, and the FYM graph **(B)** was averaged across fertilizer rates. Fertilizer was applied at 0, 17, 26, or 39 kg P ha^−1^, and FYM was applied at 0 or 10 T ha^−1^ with 0 or 15 kg P ha^−1^, respectively (FYM = 0.15% P). Treatment means with different letters are significantly different at *p<* 0.05. Data are shown as means ± SD (*n* = 3).

There were highly significant differences in SOM (**Table 4**) for both P sources (fertilizer and FYM) but not for the interaction. For fertilizer (F), combined across FYM treatments, the increase was mostly linear and significant at the two highest P rates (**Figure 4A**). For FYM (M), combined across fertilizer rates, the increase was also significant (**Figure 4B**).

There were highly significant differences in pH (**Table 4**) for fertilizer P but not FYM or the interaction. For F, combined across FYM treatments, the decrease was mostly linear for the first two fertilizer rates but then plateaued across the highest two rates (**Figure 4A**). For FYM (M), there was a trend for a pH decrease with the FYM application, but the difference was not significant (**Figure 4B**).

There were highly significant differences in plant-available P, as measured by AB-DTPA-extractable soil P (**Table 4**), for both P sources (fertilizer and FYM), but not for the interaction. Application of P alone, as well as in combination with FYM, modestly increased postharvest available P over the control, with a general increase as the P rate increased (**Table 5**). The highest P rate resulted in a significant increase over the control (*p*< 0.05).

### 3.5 PCA and correlation: Field experiment

The PCA was computed to detect interrelationships among measured traits and to determine the importance of the measured traits on the evaluation of grain yield and P efficiency; the PCA was conducted using the experimental dataset including all eight treatments and 11 variables to reduce the dimensionality of the data and to reveal the potential relationships among the measured traits. The PCA results identified that the first two principal components (PCs) with eigenvalues of >1 were accounted for (**Table 6**). The measured traits are appropriate for accounting 94.34% of the total variance: PC1 (84.04%) and PC2 (10.33%). The PC1 was mainly explained by: SOM, pH, grain yield (GY), straw (SY), plant-available P (AB-DTPA), PU, PB, P Transf, and week T. The PC2 showed a high correlation with only PAE and PUE.

**Table 6:** Field study: Principal component analysis (PCA) of the selected trials showing the amount of variance explained by individual principal components (PC) with PCA values.

Component	PC1	PC2
Eigenvalue	9.24	1.14
Variability %	84.04	10.33
Cumulative %	84.04	94.37
Parameters	Factor loadings	
SOM	0.837^a^	0.505
pH	−0.756^a^	−0.601
Grain yield	0.755^a^	0.628
Straw yield	0.901^a^	0.397
Plant-available P (AB-DTPA)	0.968^a^	0.189
PU	0.835^a^	0.513
PUE	0.063	0.969^a^
PAE	0.676	0.728^a^
PB	0.937^a^	0.253
P Transf	0.949^a^	0.201
Week T	0.885^a^	0.175

a Traits for the suggested factor.

**Figure 5:**
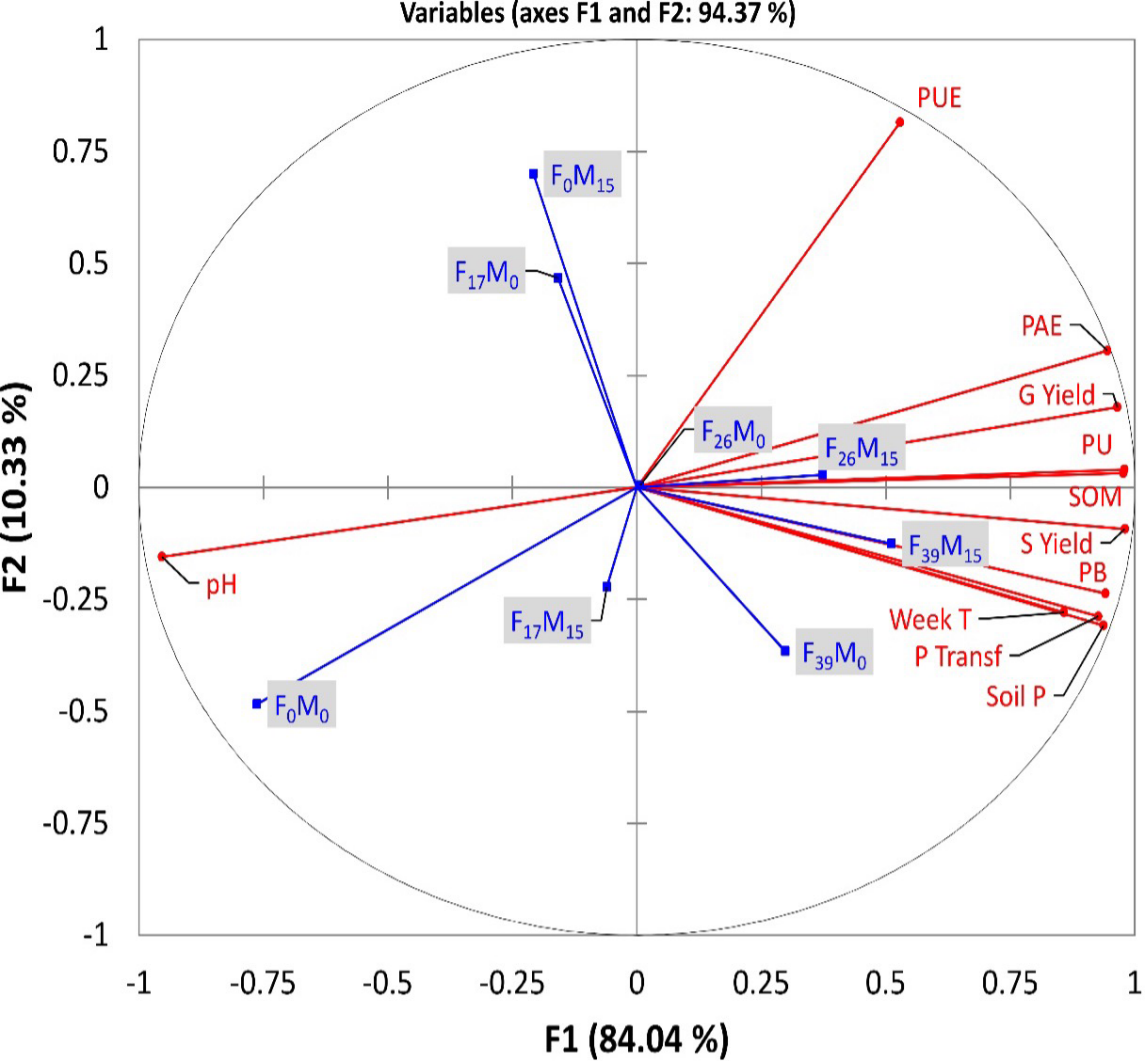
Principal component analysis (PCA) indicating the impact of treatments and their relationships with various soil properties and yield. Treatments (in blue font) are described in **Table 1**. Measured parameters (in red font) include phosphorus **(P)** use efficiency (PUE), P acquisition efficiency (PAE), grain yield (G yield), P uptake (PU), soil organic matter (SOM), straw yield (S yield), P balance (PB), and plant-available P (AB-DTPA) (soil P).

Grain yield and straw yield were significantly correlated to SOM, PU, and PAE (**Figure 5**). Moreover, the superimposition of various treatments on the variable plot showed that wheat treated with the F_26_M_15_ and F_39_M_15_ treatments represented a higher correlation with soil P, weekly turnover, PB, SOM, PU, straw, and grain yield (**Figure 5**). In contrast, fertilizer-only P (up to 26) or FYM application showed a negative association with various parameters.

**Figure 6:**
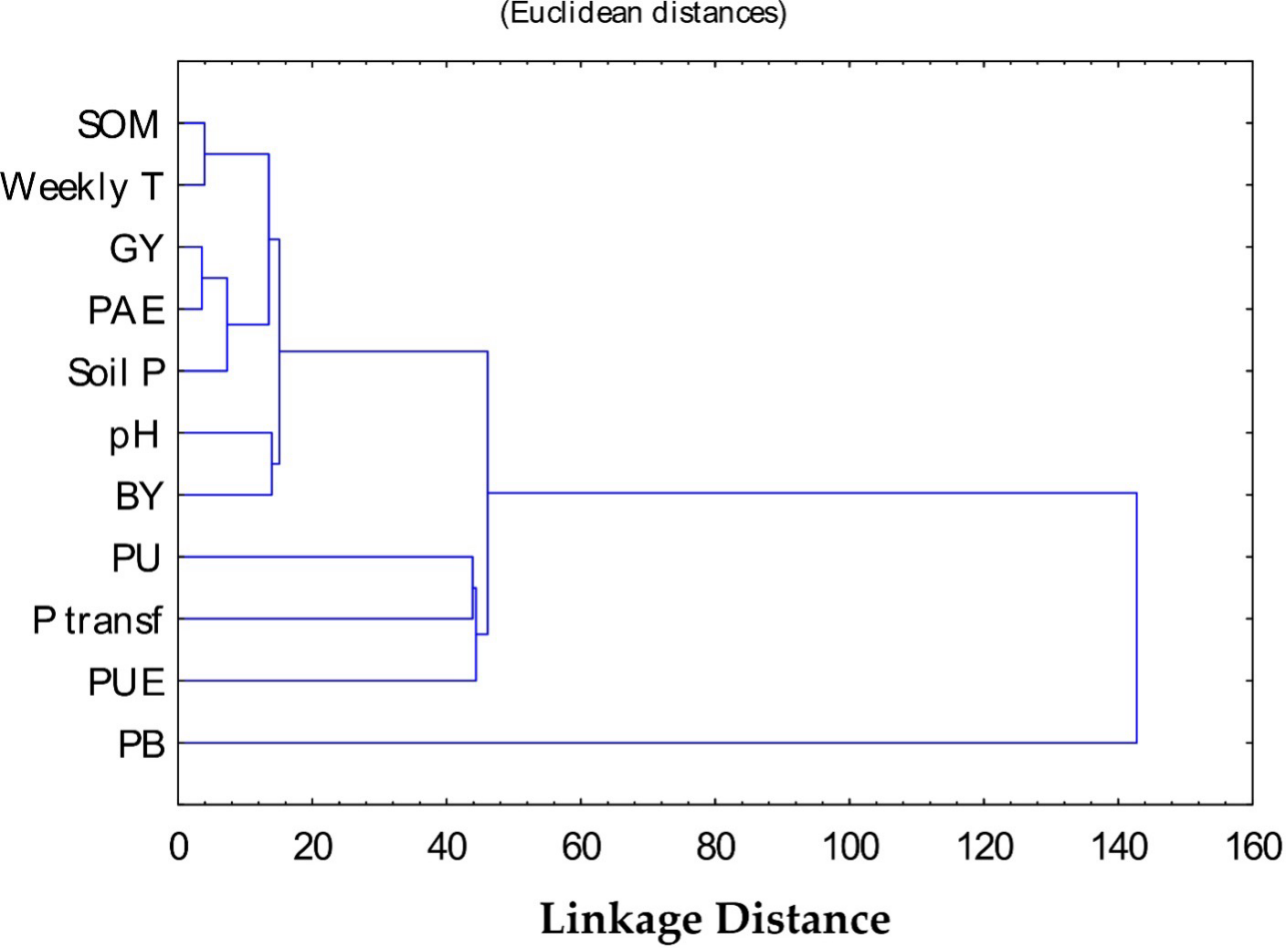
Cluster analysis (CA) of 11 traits of soil and wheat grown under calcareous conditions. Measured parameters include soil organic matter (SOM), grain yield (GY), P acquisition efficiency (PAE), plant-available P (AB-DTPA) (soil P), P uptake (PU), and phosphorus **(P)** use efficiency (PUE).

The information obtained from the application of PCA allowed the identification of the most important traits related to wheat yield. The PCA conferred the positive effects of mineral-P concoction with FYM on SOM, soil P, PU, and wheat yield. This notation is further sustained by the CA results, which revealed that most traits of the PC1 were located in the same group (cluster I) (**Figure 6**). This suggests that those traits clustered together could contribute the most to influencing wheat yield under our study conditions.

**Figure 7:**
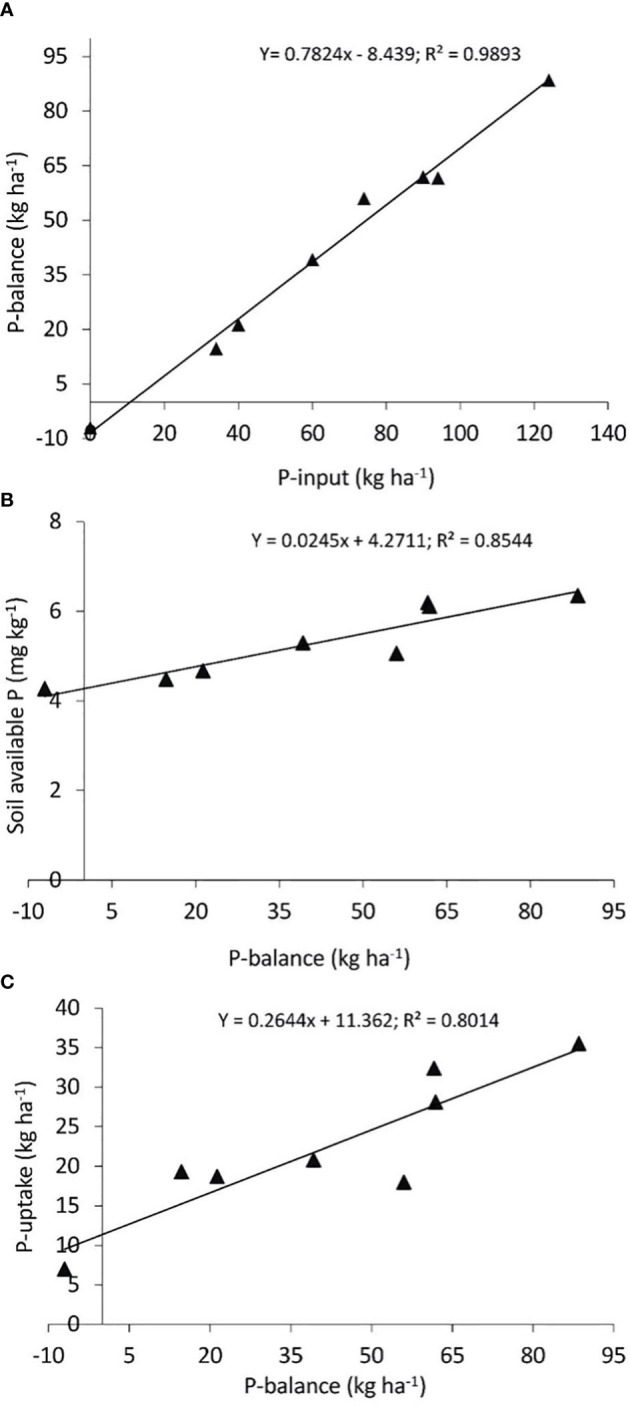
Simple linear regression analysis shows the relationship of P balance (PB) with P input **(A)**, soil (plant)-available P as AB-DTPA with PB **(B)**, and P uptake with PB **(C)**.

A strong positive regression coefficient (*y* = 0.782*x* − 8.439, *R* ^2^ = 0.98) was observed between total P input and PB (**Figure 7A**). Positive values of PB are similar to the accumulation of P in soil and are good for soil fertility improvement, while negative values (such as for the zero P rate) indicate crop P starvation and suggest that the soil is being “mined” of soil P. This may result in a reduction in fertility (exhaustion). Similarly, there was a strong correlation between PB with plant-available P (AB-DTPA) (*y* = 0.024*x* + 4.271, *R* ^2^ = 0.84) (**Figure 7B**) and PU in plants (*y* = 0.2644*x* + 11.362, *R* ^2^ = 0.80) (**Figure 7C**). These observations indicate that PB is directly linked with the external application of P, which not only increased plant-available P (AB-DTPA) but was also helpful in the assimilation of P in growing plants.

## 4 Discussion

### 4.1 Plant-available P and mineralization potential

We generally observed a slow and gradual increase in plant-available P (AB-DTPA) with the addition of fertilizer P and FYM, which increased further with the incubation time. In general, FYM application has appreciable and dynamic impacts on the chemical fractions of P because P from FYM gradually turns into available forms over time (Hopkins, 2015; Ma et al., 2020).

It has been reported by Amin (2018) that the addition of FYM to calcareous soils significantly increased plant-available P (AB-DTPA). The increase in available P in our study might be due to the release of significant quantities of CO_2_ during FYM decomposition (Andriamananjara et al., 2019) and the complexing of cations such as Ca^+2^, thus reducing P fixation in calcareous soils (Lindsay, 2001; Fixen and Bruulsema, 2014; Jamal et al., 2018). The FYM contains organic acids, which are known to complex P and increase its solubility (Hill et al., 2015a; Hill et al., 2015b; Hopkins, 2015; Summerhays et al., 2015). Furthermore, FYM application, in conjunction with fertilizer P, increased the plant-available P throughout the incubation period. The application of animal manure, similar to the FYM used in this study, may increase the bioavailability of soil P by improving the concentrations of soil-dissolved organic carbon (Jamal et al., 2018). Our results are in line with the study of Yan et al. (2016), who reported that manure application increased the proportion of plant-available P due to the transformation of stable P to labile P.

In our study, the mineralization potential was found to increase with increasing levels of fertilizer P. There is also some evidence of it increasing with FYM application, but only at the 0- and 26-kg P ha^−1^ fertilizer rates. However, the highest mineralization potential was achieved at the highest fertilizer P rate without FYM application, being significantly greater than all other treatments. Amin (2018) also found that high levels of FYM addition enhanced P mineralization in calcareous soils.

### 4.2 Postharvest soil properties

The application of fertilizer P generally increased SOM (**Figure 4A**). The addition of FYM also increased SOM. The increase in SOM with FYM treatment may be partially due to the input of organic matter found in the FYM (Rehim et al., 2020), although the increase in SOM with chemical fertilizer would not be explained by this as it contains no organic material. Rather, this increase is likely related to increased plant biomass in the current year’s crop.

Other researchers have found that FYM not only reduced the oxidation stability of SOM but also improved the SOM content of the soil up to 1.2–2.9 kg ha^−1^ (Li et al., 2017; Ding et al., 2020). Furthermore, the addition of FYM with a combination of inorganic fertilizers to soil has been reported to increase the efficiency of applied fertilizers (Elgharably, 2020). Moreover, the addition of FYM with inorganic fertilizers can increase SOM and consequently soil water and nutrient holding capacity (Urbaniak et al., 2017).

The addition of FYM did not significantly decrease soil pH in our study, but it trended downward. Rehim et al. (2020) found that the addition of FYM decreased soil pH in calcareous sandy soil. The soil pH could have been reduced due to the chemical oxidation and microbiological decomposition of FYM in soil, which produced acidic compounds that help reduce soil pH. The production of organic acids (amino acid, glycine, cysteine, and humic acid) during the mineralization (ammonization and ammonification) of organic materials by heterotrophs and nitrification by autotrophs can also cause a decrease in soil pH (Kumar et al., 2020). We did measure a significant decrease in pH with the application of SSP fertilizer. During the mineralization and chemical transformations of organic and inorganic fertilizers, the H^+^ ions released can decrease soil pH if the soil is not highly buffered with carbonates or similar substances (Hopkins, 2015; Ding et al., 2020).

The combined application of P fertilizers and organic amendments significantly increased P accumulation in soil, which agrees with the findings of various researchers (Hopkins, 2015; Qaswar et al., 2020; Rehim et al., 2020; Pradhan et al., 2021; Mihoub et al., 2022). This may be due to low levels of P in the soils and inputs from both organic and inorganic P sources (Shah et al., 2013).

Postharvest plant-available P (AB-DTPA) significantly increased with the application of fertilizer P. The addition of FYM increased this further, but only at one rate (possibly due to the great PU in the plant and less remaining in the soil). This effect may be attributed to the release of both P and low molecular weight organic acids during organic amendment decomposition (Pradhan et al., 2021). The organic acids/anions can dissolve insoluble P and compete with phosphate for adsorption sites on the surfaces of soil particles, thus increasing P availability (Jamal et al., 2018). Furthermore, organic amendments enhance soil biological and enzyme activities and increase organism abundance, thus enhancing P availability through dissolved organic carbon in the soil (Wu et al., 2013). The studied soil was highly calcareous, and the Ca–P was the most abundant fraction in these soils due to the high content of Ca^2+^ that forms calcium phosphates with P and hampers the availability of P (Saeed et al., 2021). The organic compounds can decrease the formation of Ca–P due to calcium solubilization by decreasing the soil pH due to the release of organic acids from SOM decomposition (Kumar et al., 2020). Additionally, these molecules, found in manure and similarly in ancient deposits of formerly living materials (e.g., Leonardite), or even with manmade molecules, can complex P to improve its solubility and mobility (Hopkins, 2013; Hill et al., 2015a; Hill et al., 2015b; Hopkins, 2015; Summerhays et al., 2015; Hopkins et al., 2018). For these reasons, manures can improve soil P availability and uptake by plants.

### 4.3 Yields and P Efficiency

Maximum wheat grain yield was achieved at the highest rate of P fertilizer (39 kg P ha^−1^), regardless of whether FYM was applied or not. However, an equivalently high yield was achieved at the next lower rate of P fertilizer (26 kg P ha^−1^) when FYM was also applied. Similarly, the findings of Ibrahim et al. (2008) revealed that FYM and different levels of compost have significantly increased wheat grain yield due to improved soil physical conditions, enhanced soil fertility, and improved stand establishment.

In the present study, the uptake of P by wheat was increased with P fertilizer and FYM. The increased uptake of P by wheat at an increased level of chemical P fertilizer and FYM could be due to the fact that FYM releases more nutrients over time, so that nutrient loss is less, which might have resulted in more plant PU. Furthermore, it could be due to the balanced and steady supply of nutrients to plants at all stages of crop growth.

Efficiencies for P, such as PAE and PUE, were determined in this study. PAE denotes the aptitude of crops to take up P from the soil, and PUE is the ability to produce biomass using the acquired P. Improving P efficiency can be reached by improving the acquisition and utilization of P (Parentoni and Lopez de Souza, 2008). In general, inorganic P caused an increase in PUE when applied in combination with FYM; however, PUE values decreased at higher FYM application rates. These results were in agreement with the findings by Rahim et al. (2010) and Castillo et al. (2013), who found that the PUE of wheat decreased significantly at a higher P rate.

We observed positive PB for all the treatments except the control. This shows that even the low levels of P fertilizer used in the present study resulted in a building of residual soil P. It has been reported by many that application of FYM alone or in combination with inorganic fertilizer increases the soil nutrient balance (Blake et al., 2000; Meena et al., 2017; Pradhan et al., 2021). Furthermore, we observed (**Figure 3**) strong correlations between P input vs. PB (*R* ^2^ = 0.98), PB vs. plant-available P (AB-DTPA) (*R* ^2^ = 0.84), and PB vs. PU (*R* ^2^ = 0.80). The PCA analysis also clustered various plant growth parameters with P26 and P39 along with FYM and conferred positive effects of applied treatments on soil properties and wheat yield (**Figure 5**). These positive linear relationships might be due to the cause of positive PB in these soils (Tang et al., 2008). These values have been successfully used as a tool for predicting the change in the soil P status and recommending the amount of P application practically (Blake et al., 2000). Plant-available P (AB-DTPA) in these highly alkaline calcareous soils has very low availability due to high fixation, and slow diffusion, thus limiting plant growth and crop yield. The addition of FYM alone or in combination with inorganic fertilizers decreases P fixation owing to the inactivation of Fe and Al ions thus improving the adsorption capacity of P in soil (Jamal et al., 2018; Saeed et al., 2021).

We note that, in terms of environmental considerations, the highest PUE was at the lowest rate of P (F_0_M_15_). However, this rate does not enable sufficient overall sustainability as the yields were so low. Agricultural producers have the responsibility to provide food, fuel, and fiber for the eight billion people on Earth. Low yields result in an increase in the amount of arable land. Thus, a combination of reasonable yields along with good P efficiency is a vital consideration. In our study, the second highest PUE was at the statistically highest grain and straw yield, providing for farm, environmental, and societal sustainability.

## 5 Conclusion

The SOM increased linearly for both fertilizer and FYM, whereas the pH decreased with fertilizer only. The addition of fertilizer and FYM increased plant-available P by an average of 0.5 mg P kg^−1^ soil week^−1^ with incubation. With this, plant-available P (AB-DTPA) increased initially (~1–9 mg P kg^−1^), with further gains by the end of the incubation (~3–17 mg P kg^−1^). In the field study, there was also a significant increase of available P in the soil supporting plants, although the magnitude of the increase was much smaller, with a maximum 2 mg kg^−1^ significant increase for the highest fertilizer rate with FYM. The increased plant-available P resulted in significant increases in grain and straw yields, which peaked with fertilizer at 26 kg P ha^−1^ plus FYM. The PU, PB, and PAE increased linearly with the P rate, with the highest levels at the highest P rate. In general, efficiency increased with FYM. The PUE was highest at the lowest rates of P, with general decreases with increasing P, although not consistently. The PCA revealed that 94.34% of the total variance was accounted for by PC1 (84.04%) and PC2 (10.33%), with grain straw yield significantly correlated to SOM, PU, and PAE. A strong positive regression coefficient was observed between PB and total P input, plant-available P (AB-DTPA), and PU. The outcomes of our study would help to update recommendations for P fertilizer application in calcareous soils while sustaining soil fertility and simultaneously reducing fertilizer costs and conserving limited resources.

## Data availability statement

The raw data supporting the conclusions of this article will be made available by the authors, without undue reservation.

## Author contributions

AJ: Planned research work and write-up of first drafts. MFS: Supervised the research and improved the first draft. AM: Performed conceptualization, improving draft and helped in data analysis. BH: Review, writing/improving draft. IA: Co-supervised research. AN: Helped in methodology, data curation. All authors contributed to the article and approved the submitted version.
