# Supplementary material for: Integrated use of phosphorus fertilizer and farmyard manure improves wheat productivity by improving soil quality and P availability in calcareous soil under subhumid conditions
Source: Front Plant Sci. 2023 Jan 23;14:1034421. doi: 10.3389/fpls.2023.1034421 (PMC9900179; doi:10.3389/fpls.2023.1034421)
Supplement: Supplementary file 1 [file DataSheet_1.pdf]

Table S1. Incubation Study: Statistics for P transformations.

	SS	DF	MS	F value	P value
Intercept	20902.41	1	20902.41	86170.10	<0.001
Incubation period	504.83	5	100.97	416.24	<0.001
Fertilizer	1537.56	3	512.52	2112.87	<0.001
Manure	58.40	1	58.40	240.73	<0.001
Incubation period*Fertilizer	188.87	15	12.59	51.91	<0.001
Incubation period*Manure	19.70	5	3.94	16.24	<0.001
Fertilizer*Manure	110.10	3	36.70	151.30	<0.001
Incubation period*Fertilizer*Manure	19.11	15	1.27	5.25	<0.001
Error	23.29	96	0.24		

Table S2. Incubation Study: Statistics for the weekly turnover P.

	SS	DF	MS	F value	P value
Intercept	2.530127	1	2.530127	2030.541	<0.001
Treatments	0.838948	7	0.119850	96.185	<0.001
Error	0.019937	16	0.001246		

Table S3. Incubation Study: Statistics for the mineralization potential of P.

	SS	DF	MS	F value	P value
Intercept	5829.4134	1	5829.4134	2030.5413	<0.001
Treatments	1932.93593	7	276.133705	96.1847891	<0.001
Error	45.9338667	16	2.87086667		
